# Lipid Modulation in the Formation of β-Sheet Structures. Implications for De Novo Design of Human Islet Amyloid Polypeptide and the Impact on β-Cell Homeostasis

**DOI:** 10.3390/biom10091201

**Published:** 2020-08-19

**Authors:** Israel Martínez-Navarro, Raúl Díaz-Molina, Angel Pulido-Capiz, Jaime Mas-Oliva, Ismael Luna-Reyes, Eustolia Rodríguez-Velázquez, Ignacio A. Rivero, Marco A. Ramos-Ibarra, Manuel Alatorre-Meda, Victor García-González

**Affiliations:** 1 Departamento de Bioquímica, Facultad de Medicina Mexicali, Universidad Autónoma de Baja California, Mexicali 21000, Baja California, Mexico; israel.martinez.navarro@uabc.edu.mx (I.M.-N.); rauldiaz@uabc.edu.mx (R.D.-M.); pulido.angel@uabc.edu.mx (A.P.-C.); 2 Laboratorio de Biología Molecular, Facultad de Medicina Mexicali, Universidad Autónoma de Baja California, Mexicali 21000, Baja California, Mexico.; 3 Instituto de Fisiología Celular, Universidad Nacional Autónoma de México, Ciudad de Mexico 04510, Mexico; jmas@ifc.unam.mx (J.M.-O.); ismaelluna.biome@gmail.com (I.L.-R.); 4 Facultad de Odontología, Universidad Autónoma de Baja California, Tijuana 22390, Mexico; eustolia.rodriguez@uabc.edu.mx; 5 Tecnológico Nacional de México/I.T. Tijuana, Centro de Graduados e Investigación en Química-Grupo de Biomateriales y Nanomedicina, Tijuana 22510, Mexico; 6 Tecnológico Nacional de México/Instituto Tecnológico de Tijuana, Centro de Graduados e Investigación en Química, Tijuana 22510, Baja California, Mexico; irivero@tectijuana.mx; 7 Facultad de Ciencias Químicas e Ingeniería, Universidad Autónoma de Baja California, Tijuana 22390, Baja California, Mexico; mramos@uabc.edu.mx; 8 Cátedras CONACyT- Tecnológico Nacional de México/I.T. Tijuana, Centro de Graduados e Investigación en Química-Grupo de Biomateriales y Nanomedicina, Tijuana 22510, Mexico; manuel_alatorre@yahoo.com.mx

**Keywords:** hIAPP, amyloidogenesis, insulin granules, endoplasmic reticulum, anionic lipids, F_23_R variant, β-sheet transitions, β-cell cytotoxicity, unfolded protein response

## Abstract

Human islet amyloid polypeptide (hIAPP) corresponds to a 37-residue hormone present in insulin granules that maintains a high propensity to form β-sheet structures during co-secretion with insulin. Previously, employing a biomimetic approach, we proposed a panel of optimized IAPP sequences with only one residue substitution that shows the capability to reduce amyloidogenesis. Taking into account that specific membrane lipids have been considered as a key factor in the induction of cytotoxicity, in this study, following the same design strategy, we characterize the effect of a series of lipids upon several polypeptide domains that show the highest aggregation propensity. The characterization of the C-native segment of hIAPP (residues F_23_-Y_37_), together with novel variants F_23_R and I_26_A allowed us to demonstrate an effect upon the formation of β-sheet structures. Our results suggest that zwitterionic phospholipids promote adsorption of the C-native segments at the lipid-interface and β-sheet formation with the exception of the F_23_R variant. Moreover, the presence of cholesterol did not modify this behavior, and the β-sheet structural transitions were not registered when the N-terminal domain of hIAPP (K_1_-S_20_) was characterized. Considering that insulin granules are enriched in phosphatidylserine (PS), the property of lipid vesicles containing negatively charged lipids was also evaluated. We found that these types of lipids promote β-sheet conformational transitions in both the C-native segment and the new variants. Furthermore, these PS/peptides arrangements are internalized in Langerhans islet β-cells, localized in the endoplasmic reticulum, and trigger critical pathways such as unfolded protein response (UPR), affecting insulin secretion. Since this phenomenon was associated with the presence of cytotoxicity on Langerhans islet β-cells, it can be concluded that the anionic lipid environment and degree of solvation are critical conditions for the stability of segments with the propensity to form β-sheet structures, a situation that will eventually affect the structural characteristics and stability of IAPP within insulin granules, thus modifying the insulin secretion.

## 1. Introduction

Human islet amyloid polypeptide (hIAPP) is a hormone that slows down gastric emptying and participates in the regulation of plasmatic glucose associated with functions such as glucagon-release inhibition and leptin sensitization [1,2]. hIAPP is a monomeric peptide processed in the Golgi complex and secreted in insulin granules in response to β-cell secretagogues [3]. However, amyloid fibril formation in hIAPP could trigger an amplified toxicity response that leads to failure of pancreatic β-cells, a hallmark of type 2 diabetes mellitus (T2DM). Several variants of hIAPP modify their stability accelerating amyloid formation; for instance, in Asian populations, the S_20_G mutation has been associated with early-stage cases of DM2 [4]. Likewise, variants have been described in the Maori populations of New Zealand [5]. By contrast, rat IAPP (rIAPP) containing structural differences with hIAPP in six residues situated in region 18–29 (H_18_R, F_23_L, A_25_P, I_26_V, S_28_P, and S_29_P) show a diminished trend to produce amyloid fibrils [6]. Although these changes allowed the development of pramlintide, an amylinomimetic peptide with three variants (A_25_P, S_28_P, and S_29_P) used in DM2 therapy; the propensity to aggregation of this sequence is not completely avoided.

Several reports suggest that the C-native segment of IAPP (residues 23–37) is a critical domain in the structural transitions that trigger amyloid formation [6], hence, it could be a target for the development of strategies to reduce aggregation. Based on multiple sequence alignment among N- and C-domains on 240 sequences of different species, the N-domain (residues 1–20) has been described as a conserved sequence among a wide variety of organisms, while the C-domain (residues 22–37) has been restricted to phylogenetically close groups [7]. Therefore, we proposed a panel of optimized IAPP sequences, which could reduce aggregation with only one residue substitution [7].

Localized changes in the secondary structure of proteins and peptides are believed to work as a molecular switch regulating function or, in some cases, as a trigger for misfolding. In this context, we have described these conditions by studying the cholesteryl-ester transfer protein (CETP) and a series of apolipoproteins [8,9,10,11,12,13]. In addition, other studies have reported that phosphatidylserine (PS) vesicles could increase the peptide/aggregation ratio, suggesting an electrostatic factor as a triggering condition for the induction of conformational changes [14,15]. In this sense, within insulin secretory granules derived from the endoplasmic reticulum (ER) [16], the concentration of PS and phosphatidylinositol has been described to be fivefold higher compared to that of the cell membrane [17]. Therefore, lipids could be a factor that promotes structural changes in hIAPP, inducing misfolding phenomena.

However, highly sophisticated mechanisms that modulate protein structure and function have evolved to maintain cellular homeostasis and counteract misfolding [11]. Perturbations in these mechanisms can lead to protein dysfunction as well as deleterious cell processes. Specifically, imbalances in secretory protein synthesis pathways lead to a condition known as ER stress, which elicits the adaptive unfolded protein response (UPR) [11], a phenomenon that could be critical during insulin maturation. Importantly, proinsulin is folded in the ER by chaperones such as protein disulfide isomerases (PDI) and binding immunoglobulin protein (BiP) or GRP-78 [18]. Therefore, the transducers of the UPR pathway, IRE1, ATF6α, and PERK, promote the translation of target chaperones through XBPIs, ATF6α, and CHOP transcription factors, respectively, when unfolded proteins accumulate in the lumen [19]. The three branches of UPR are essential in cell homeostasis to reduce ER stress and to ensure adequate synthesis of peptides such as insulin [20]. Indeed, in several reports, we have described the effect of metabolic overload on the dysregulation of UPR arms [21,22].

Given this situation, the initial events of misfolding and amyloid aggregation promote a cascade of pathological processes considered the hallmark in the progression of several chronic degenerative diseases [8,9,10,11,12,13] that might be associated with conditions related to metabolic overload [21,22]. Having this in mind, we herein characterized the role of lipid systems on the conformational transitions of the most aggregative C-terminal domain of hIAPP, and through a biomimetic approach, evaluated this condition on variants F_23_R and I_26_A that potentially could reduce aggregation. In addition, we also characterized the association with cell responses involved in protein homeostasis such as UPR.

## 2. Materials and Methods

### 2.1. De Novo IAPP Sequences

Based on network analysis, different residues from 240 species reported in the NCBI database were replaced on the hIAPP sequence. The effect of the substitutions was characterized through physicochemical assays, as well as by the computational identification of regions with a high intrinsic propensity for aggregation [7]. Aggregation propensity range was obtained considering the aggregation value of hIAPP as a reference, based on the AGGRESCAN algorithm (Na4vSS) [23].

### 2.2. Materials

Cell culture reagents were purchased from Thermo Fisher (Carlsbad, CA, USA), while tissue culture plates and other plastic materials were obtained from Corning Inc. (Corning, NY, USA). Salts and buffers were obtained from Sigma-Aldrich (St. Luis, MI, USA), as well as Thioflavin T (ThT), black Sudan B, Congo red, sodium dodecyl sulfate (SDS), and 3-(4,5-dimethylthiazol-2-yl)-2,5-diphenyltetrazolium bromide (MTT). l-α-phosphatidylcholine (PC), l-α-phosphatidylserine (PS), l-α-phosphatidyl-ethanolamine (PE), 1-oleoyl-2-hydroxy-sn-glycero-3-phosphate (LPA), 1-palmitoyl-2-oleoyl-sn-glycerol (POPG), and cholesterol were obtained from Avanti Polar Lipids, Inc. (Alabaster, AL, USA). Antibodies anti-XBP1s and anti-BiP/GRP78 were purchased from Abcam (Cambridge, UK) and anti-β-actin was obtained from Santa Cruz Biotechnology (Dallas, TX, USA). Anti-PDI was donated by Dr. Marco A. Ramos Ibarra.

### 2.3. Peptide Synthesis and Preparation

**Figure 1:**
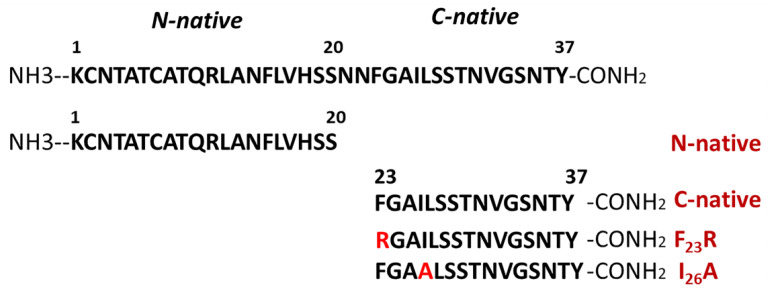
Representation of the primary structure of human islet amyloid polypeptide (hIAPP) and peptides corresponding to N-native and C-native domains, as well as new variants of hIAPP.

Several peptides were synthesized considering the physicochemical properties of hIAPP such as: N-native segment (^1^KCNTATCATQRLANFLVHSS^20^); C-native segment (^23^FGAILSSTNVGSNTY^37^); F_23_R variant (^23^RGAILSSTNVGSNTY^37^); and I26A variant (^23^FGAALSSTNVGSNTY^37^) (Figure 1). Likewise, the aggregative core of amyloid beta (Aβ) peptide was used as a control ^25^GSNKGAIIGLM^35^. All solutions were filtered through 0.22 μm membrane filters (Millipore, Burlington, MA, USA) before the experiments. Peptide purity greater than 98% was confirmed by mass spectrometry and HPLC (GenScript, Piscataway, NY, USA). The best condition for peptide solubilization was the use of ultrapure H_2_O (600 µg/mL), subsequently diluted in phosphate buffer pH 7.4 (60 µg/mL).

### 2.4. Preparation of Small Unilamellar Vesicles (SUVs)

SUVs were prepared from PC, PS, PE, cholesterol, and POPG (600 µg/mL) upon their mixing and conditioning at varying concentrations. PC, PS, PE, cholesterol, and POPG were dissolved in chloroform and dried for 90 min under a gentle stream of N_2_ with an additional incubation of 5 h at 30 °C in an Eppendorf Vacufuge concentrator (Eppendorf, Hamburg, Germany), according to protocols established by our working group [9]. After drying, samples were hydrated in phosphate buffer to pH 7.4 and processed through 4 cycles of freezing in liquid N_2_ and thawing at 37 °C, and finally subjected to a sonication process (for 10 min under 15 s on/30 s off cycles at 9.5–10 W) in a Cole-Parmer Ultrasonic Homogenizer (Vernon Hills, IL, USA). Samples were stabilized for 1 h at 25 °C and centrifuged at 13,000 rpm for 10 min.

LPA samples in chloroform were placed under a gentle flow of N_2_ for 90 min and additional 12 h in a vacuum equipment. The samples were hydrated in phosphate buffer and afterwards processed through 4 cycles of freezing in liquid N_2_ and thawing at 37 °C. Solutions were left to equilibrate for 2 h and centrifuged at 13,000 rpm for 10 min.

### 2.5. Preparation of Large Unilamellar Vesicles (LUVs)

LUVs were prepared from PC and PS by the reverse-phase evaporation methodology [24], with some adaptations. Specifically, PC LUVs were prepared by dissolving the lipid (600 µg/mL) in a 1:3 mixture of diethylether:phosphate buffer (pH 7.4). Then, the solution was sonicated for 5 min in an ultrasonic homogenizer. Finally, the solution was added to a rotary evaporator working at an initial pressure of 400 mmHg for 5 min, followed by a final pressure of 650 mmHg until complete removal of diethylether. Likewise, PS LUVs were prepared by dissolving the lipid (600 µg/mL) in chloroform and drying the resulting solution for 90 min under a gentle stream of N_2_ with an additional incubation of 2 h at 30 °C in an Eppendorf Vacufuge concentrator, according to protocols established by our working group [9]. After drying, samples were hydrated in phosphate buffer, pH 7.4, and processed through 4 cycles of freezing in liquid N_2_ and thawing at 37 °C. Finally, samples were subjected to a sonication process (for 5 min under 15 s on/30 s off cycles at 9.5–10 W) in an ultrasonic homogenizer.

### 2.6. Dynamic Light Scattering Experimentation and Optical Density Characterization

In the characterization of vesicle size, PC (300 µM) and PS (300 µM) vesicles were evaluated. The hydrodynamic diameter (Dh) and Z-Potential of the vesicles were assessed by dynamic light scattering (DLS), employing a Zetasizer Nanoseries spectrophotometer (Malvern Instruments, Malvern, UK). All measurements were carried out at 25 °C using polystyrene disposable cells and folded capillary cells for the size and ζ potential measurements, respectively. Before their characterization, the samples were vortexed for 10 s and left to rest for 30 min at 25 °C. The results are the average of five measurements. In another batch of vesicles (PS), we completed the characterization by using a Microtrac equipment. In a complementary way, we performed the optical density characterization of vesicles employing a BioRad Smart spectrophotometer with diode array (Hercules, CA, USA).

### 2.7. Peptide Bond Conformational Changes

Experiments were performed through the characterization of optical density at 218 nm, which is associated with conformational changes along the formation of β-sheet structures [8,10]. The effect of lipid vesicles composed of several lipids on conformational changes of hIAPP-derived peptides was evaluated. Measurements were obtained using the above-described BioRad Smart spectrophotometer, employing a peptide concentration of 60 µg/mL and then evaluating the effect of the lipid vesicles.

### 2.8. Congo Red Birefringence Spectroscopy

Assays were performed based on a previous protocol [8], employing 10.6 μM Congo red and 60 μg/mL peptides solutions. The optical density was measured at 494 nm, employing the above-described BioRad Smart spectrophotometer, under varying solution conditions.

### 2.9. ThT-Fluorescence Assay

β-sheet structures of peptides were characterized through the ThT-fluorescence assay. Samples were incubated for 12 h at 37 °C and monitored with the ThT (20 μM) treatment. Fluorescence emission spectra were registered at 25 °C from 460 to 610 nm with an excitation wavelength of 450 nm in a Cary Eclipse Fluorescence spectrophotometer (Agilent Technologies, Inc., Santa Clara, CA, USA).

### 2.10. Circular Dichroism (CD)

CD experiments were performed at a peptide concentration of 120 μg/mL in a 1-mm path length quartz cuvette, using the CD neural network (CDNN) based software. Spectra were recorded with a 1-mm bandwidth, using 1 nm increments and 2.5 s accumulation time. CD spectra were recorded with an AVIV 62DS spectropolarimeter (AVIV Instruments, Lakewood, NJ, USA) at 25 °C employing far UV wavelengths (190–260 nm). CD results were reported as mean molar ellipticity (deg cm^2^ dmol^−1^).

### 2.11. Lipid–Peptide Interactions

Lipid/peptide samples were analyzed with a nondenaturing electrophoresis technique adapted by our group for lipid–peptide characterization. We established a new methodology through the use of 0.8–15% native gradient gel electrophoresis [8]. Later, gels were stained following the Sudan black and silver nitrate protocols [8].

### 2.12. Molecular Dynamics

Peptide-membrane systems were generated with the CHARMM-GUI input generator. For all systems, a MARTINI force field for polarizable amino acids and water was used. In our assays, we used the peptides in a simple lipid bilayer system, each lipid bilayer consisting of a homogenous array of 8 × 8 lipid molecules of PS (DIPS 18:2–18:2) or PC (3:1, DPOC 16:1–18:1 and POPC 16:0–18:0). Peptide models of N-native, C-native, and variants F_23_R and I_26_A were generated using the ITASSER online server; for all peptides, we used the predicted model with highest TM value. The systems were minimized using steepest descent and conjugate gradient methods. Then, five equilibration steps were performed for each system. Simulations were conducted in a 1:1 peptide-membrane system during 3000 ns at 303.15 K and 1 atm pressure. For each system, we evaluated the lipid bilayer lateral displacement from lipids as an indicator of bilayer fluidity. Our analysis was based on the GROMACS built-in function for MSD analysis. To this end, we used the built-in functions of GROMACS, which are calculated using the following equation:

$$D_{A}t=\frac{1}{6}\underset{t\to \infty }{\lim}{<∥r_{i}\left(t\right)-r_{i}\left(0\right){∥}^{2}>}_{i\in a}$$

where **r*_i_*** (***t***) indicates molecule position a t time, **r*_i_*** (**0**) indicates the position at molecules at time zero, and is calculated for molecules included in ***A*** set of molecules. Additionally, **6*D_A_^t^*** represents diffusion coefficient over time (***t***) for ***A*** set of molecules, using Einstein correlation adjusted for long simulations.

In this sense, the analysis along the membrane was performed in one single plane. Before analysis of MSD, periodic boundary conditions were converted used built-in conversion functions to ensure the continuous trajectory of molecules.

In a complementary way, we performed molecular dynamics reruns, started from previously equilibrated systems that were used in original reported simulations. The systems were evaluated under the same conditions of temperature, pressure, and number of molecules. The simulation time was reduced to 30,000 ps; 100 consecutive simulations were performed for each system where the previous simulation was used as a starting point for the subsequent one. All trajectories obtained from short simulations were joined using built-in functions of GROMACS to reach the same simulation time reported previously; the resulting trajectory file was converted into a continuous trajectory file. MSD analysis for this system is reported in Figures S3 and S6. This strategy was based on previous reports [25,26].

### 2.13. Cell Culture

β-cell line RIN-m5F (American Type Culture Collection) was grown using RPMI-1640 culture medium supplemented with 10% fetal bovine serum, 10 U/mL penicillin, 10 µg/mL streptomycin, and 25 µg/mL amphotericin B. Cultures were maintained at 37 °C in a humidified atmosphere of 95% air and 5% CO_2_.

### 2.14. Cell Viability Assay

Peptide cytotoxicity was assessed through MTT assays RIN-m5F cells, under different peptide and lipid–peptide treatments. Cells were seeded into 96-well plates at a density of 20,000 cells/well and allowed to grow to 90% of confluence. Next, the culture medium was replaced with Opti-MEM medium. After 1 h under this condition, cells were incubated under the different treatments and subsequently processed according to previous protocols [8].

### 2.15. Western Blotting Analysis

Under different peptide and PS-SUVs treatments, the expression of proteins associated with the UPR pathway and insulin folding were evaluated. After experimentation on RIN-m5F cells, proteins were extracted from cell cultures using ice-cold protein lysis buffer (150 mM NaCl, 10 mM Tris, pH 7.4, 1% Triton X-100, 0.5% NP40, 1 mM EDTA, 1 mM EGTA, 0.2 mM sodium orthovanadate, 10 mM benzamidine, 10 µg/mL leupeptin, 10 µg/mL aprotinin, and 250 µM PMSF). An average of 25 µg of protein lysates were separated on 8% SDS-PAGE electrophoresis and transferred to polyvinylidene difluoride (PVDF) membranes. The membranes were blocked with 5% nonfat milk in Tris-buffered saline 0.1% Tween-20 (TBS-T) for 1 h at 37 °C and incubated at 4 °C overnight with primary antibody (anti-XBP1s, anti-BiP/GRP78, anti-PDI, anti-SERCA2, and anti-β-actin). Following washing with TBS-T, the membranes were further incubated for 1.5 h at 37 °C with the corresponding horseradish peroxidase-conjugated secondary antibodies. Proteins were detected with the enhanced chemiluminescence reagent (Immobilon Western from Millipore, Burlington, MA, USA).

### 2.16. Endoplasmic Reticulum Isolation

ER fractions were obtained from RIN-m5F cells under several PS/peptides treatments. The methodology was based on the report of Prajapati et al. [27]. Cells were proliferated in 100 mm cell culture plates at a density of 2.3 × 10^5^ cells/mL. Cells were maintained in proliferation for 72 h to reach 95% of confluence, and later, different treatments were performed on a volume of 5 mL. Later, culture cells were washed with PBS 1x, recovered, and treated with 1 mL of homogenizer buffer (30 mM Tris–HCl pH 7.4, 225 mM mannitol, 75 mM sucrose, 0.5 mM EGTA, protease inhibitor, and 0.5% BSA). Homogenates were briefly sonicated (two cycles of 15 s on/30 s off at 9.5–10 W). Then, the homogenate was centrifuged at 630× *g* for 5 min at 4 °C. Supernatant was collected and conserved, and the pellet was newly processed by sonication and centrifuged under the same conditions. The combined supernatant was centrifuged again at 630× *g* for 5 min at 4 °C (nuclei-free lysate), and 150 µL was conserved. Then, the nuclei-free lysate fraction was centrifuged at 6300× *g* for 10 min. The supernatant was transferred into a new tube, and then, it was centrifuged at 20,000× *g* for 30 min at 4 °C. The supernatant was recovered and centrifuged at 100,000× *g* for 60 min at 4 °C, using a S140-AT 2555 rotor. The supernatant corresponds to the cytoplasm fraction, whereas the pellet corresponds to the ER fraction. Protein markers (anti-BiP, anti-PDI, anti-SERCA2, and anti-β-actin) were used to evaluate the quality of the isolations (Figure S9).

### 2.17. Fabrication of Vesicles Composed of PS (30 µM) and the Fluorescent Probe BODIPY-Leu (6 µM)

BODIPY-Leu probe was synthesized according to protocols developed by our group [22]. Then, the required quantities of l-α-phosphatidylserine and BODIPY-Leu were dissolved in chloroform and mixed vigorously to obtain a clear solution. Then, the solution was dried for 90 min under a gentle stream of N_2_ with an additional incubation of 5 h at 30 °C in an Eppendorf Vacufuge concentrator, according to protocols established by our working group [9]. After drying, samples were hydrated in phosphate buffer pH 7.4 and processed through 4 cycles of freezing in liquid N_2_ and thawing at 37 °C, and finally subjected to a sonication process (for 10 min under 15 s on/30 s off cycles at 9.5–10 W) in an ultrasonic homogenizer. Samples were stabilized for 1 h at 25 °C and centrifuged at 13,000 rpm for 10 min.

### 2.18. Confocal Microscopy

A LEICA TCS-SP8 confocal scanning biological microscope (LEICA Microsystems Heidelberg GmbH, Nussloch, Germany) was employed in the characterization of the subcellular localization of PS/BODIPY-Leu vesicles (PS 30 µM/BODIPY-Leu 6 µM). RIN-m5F cells were proliferated to 90% of confluence and treated with PS/BODIPY-Leu vesicles and hIAPP-derived peptides for 20 h. Later, culture cells were washed with HBSS buffer, and then, the ER-tracker probe (1 µM) was added and incubated for 25 min at 37 °C. Cells were washed once with HBSS buffer and fixed with 4% formaldehyde for 2 min at 37 °C and mounted for observation. Macroscopically different zones were recorded, preferentially at the center of the specimens, to depict representative images. Images were recorded at excitation/emission wavelengths of 488/495-545 and 552/562-700 nm for detection of PS 30 µM/BODIPY-Leu (green) and ER-tracker (red), respectively.

### 2.19. Insulin ELISA Assays

Cells were proliferated in 100 mm cell culture plates at a density of 2.3 × 10^5^ cells/mL. Cells were maintained in proliferation for 72 h, and later, different treatments were performed on a volume of 5 mL. Cell culture medium was recovered and centrifuged for 5 min at 5000 rpm. The supernatant medium was recovered and diluted (1/3) in PBS. Insulin concentrations were quantified with the Rat Ultrasensitive Insulin ELISA kit (80-INSRTU-E01, E10; ALPCO Diagnostics, Salem, NH, USA) through several adaptations according to manufacturer’s recommendations. Absorbance readings were performed at 450 nm, and results were reported as ng/mL.

### 2.20. Statistical Analysis

Data were expressed as mean ± SD. The statistical analyses were conducted with one-way ANOVA. In MTT assays data were expressed as mean ± SD.

## 3. Results

### 3.1. Secondary Structure Characterization of hIAPP Peptides

**Figure 2:**
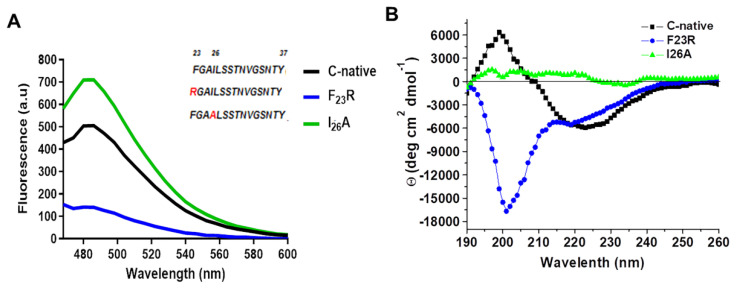
Structural characterization of C-terminal IAPP-derived peptides shows the inhibition of β-structures by the new F_23_R variant. (**A**) Fluorescence emission spectra through ThT assays on IAPP variants (60 μg/mL). The characteristic emission peak was registered at 482 nm. (**B**) Circular dichroism (CD) spectra of hIAPP variants (190–260 nm). In this representative experiment, the peptide concentration was 120 μg/mL.

In a previous report, we proposed a panel of 113 hIAPP variants, 30 of which could reduce aggregation propensity with only one substitution on the hIAPP sequence [7], compared to the pramlintide drug (3 substitutions) and several hIAPP analogs generated by substituting up to four arginine residues at positions F_23_-I_26_ [28]. Our results suggested that the 23 and 26 positions maintain secondary structure stability [7]. To assess changes in these positions, F_23_R and I_26_A variants were evaluated and compared to the highly aggregative hIAPP C-native residues (^23^FGAILSSTNVGSNTY^37^). Through ThT-fluorescence assays, the F_23_R variant showed the lowest fluorescence values corresponding to a reduced β-sheet structure formation compared with the C-native and the I_26_A variant (Figure 2A). Most probably, the result found with F_23_R is associated with the electrostatic charge of the arginine-side chain (position 23) that exerts a repulsive effect among peptide monomers.

The substitution of only F_23_R could reduce β-sheet structure formation. In the I_26_A variant, as opposed to the expected, a slightly higher signal than in the C-native segment was detected (Figure 2A). Although aggregation propensity values (AGGRESCAN Index) for I_26_A (−8.2) suggest a low tendency to generate β-structures, parameters such as hydrophobicity and isoelectric point are similar in both fragments (Table 1). Characterization of peptides was completed by CD analysis (Figure 2B), in which the C-native spectrum indicated a β-sheet structure formation. For the F_23_R variant, a peak at 190 nm and two minima at 202 and 219 nm were registered, typical of a mixture of α-helix, disordered structures, and minimal β-sheet structures. I_26_A showed an atypical behavior since the spectrum did not represent a defined secondary structure, possibly associated with amorphous aggregation.

In amyloidogenic peptides, the stability of antiparallel β-sheet structures is determined by hydrogen bonds, salt bridges, and weak polar interactions among side chains of residues [29]. Under our working conditions, the net charge of +1 for the F_23_R sequence at pH 7.4 could be a determining factor in the low propensity to aggregation, due to the likely appearance of electrostatic repulsions. In turn, I_26_A and the C-native segment showed a neutral charge at a pH range of 3.0 to 8.5 with an intrinsic tendency to aggregation, a situation that has been discussed in several reports as a problem with the therapeutics of hIAPP [30]. Therefore, a balance among the dynamic secondary structure of the hIAPP C-native segment, the net charge, and the physicochemical properties of the lipid microenvironment could define the type of the adopted secondary structure [10].

### 3.2. Effect of Lipids on the Secondary Structure of hIAPP Peptides

**Figure 3:**
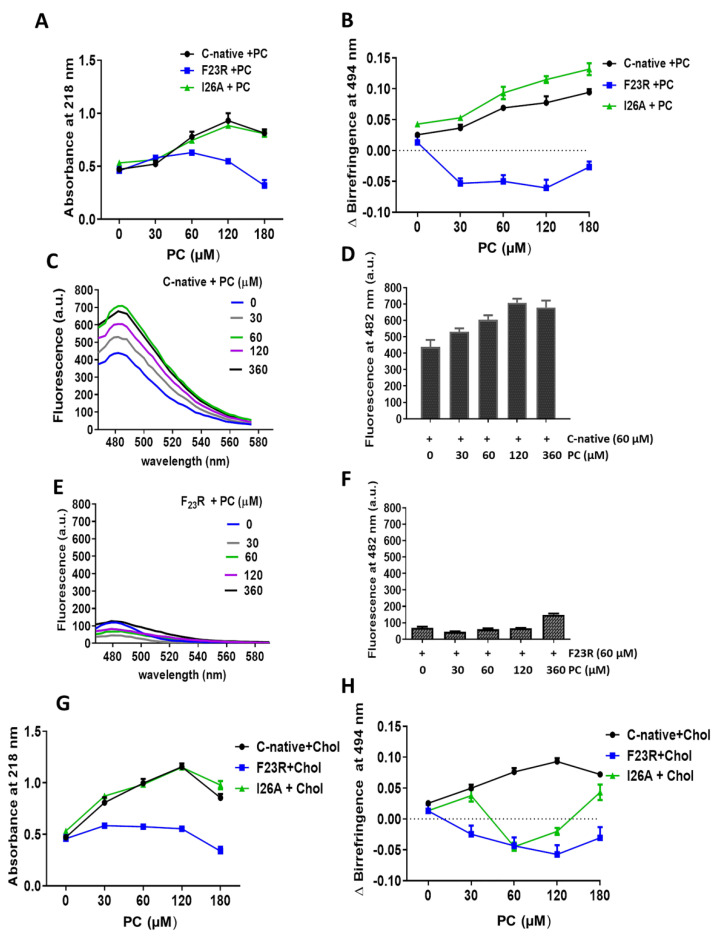
Effect of zwitterionic L-α-phosphatidylcholine-small unilamellar vesicles (PC-SUVs) on the secondary structure of IAPP-peptides. (**A**) Peptide bond absorbance of the C-native segment, F_23_R, and I_26_A under incubation with PC-SUVs. (**B**) Peptide characterization by birefringence with Congo red assay. (**C**) Interaction of the C-native segment under increasing concentrations of PC-vesicles evaluated by ThT assay. (**D**) Values of ThT-fluorescence emission at 482 nm. (**E**) Interaction between F_23_R under increasing concentrations of PC. (**F**) Values of ThT-fluorescence emission at 482 nm. Characterization of the C-native segment, F_23_R, and I_26_A under the incubation of SUVs composed of PC and cholesterol (Chol) through peptide bond absorbance at 218 nm (**G**) and Congo red assay (**H**).

The effect of lipids on the secondary structure of hIAPP peptides was characterized through the use of SUVs. In a first approach, PC-SUVs were employed as a model system and their effect on the C-native segment as well as the F_23_R and I_26_A variants was assessed through spectroscopy at 218 nm and birefringence with the Congo red assay (Figure 3). PC was chosen in the first instance since it is the most abundant zwitterionic phospholipid of the outer plasma membrane. Under incubation with increasing concentrations of PC-SUVs, the F_23_R variant did not show significant changes in the β-sheet structure content where minimal absorbance values were recorded for peptide-bond characterization. In addition, an evident difference in birefringence values compared to the C-native segment was registered (Figure 3A,B). The behavior of the I_26_A variant was comparable to the C-native segment suggesting β-sheet formation, a phenomenon associated with physicochemical parameters such as isoelectric point, hydrophobicity, and electrostatic charge (Table 1). Our data agree with the results of Cho et al., 2008, employing PC liposomes, wherein the aggregation effect of IAPP was evident [31]. By contrast, when assessing the effect of these PC SUVs on the N-native segment (^1^KCNTATCATQRLANFLVHSS^20^), β-structure formation was not found (Figure S1A–C), therefore, suggesting that structural transitions leading to β-sheet formation in hIAPP fundamentally occur on the C-native domain. Remarkably, this β-sheet structural transition exclusive to the C-native segment was likewise observed when working with PC-based large unilamellar vesicles (PC LUVs), also studied to extend the characterization of the effect of this lipid (Figure S2). While stored in β-cell granules at a pH near 5.5, the aggregation of hIAPP is inhibited; however, aggregation propensity increases when hIAPP is released into the extracellular compartment at pH 7.4 [32]. Taking into account that the effect of lipids was studied at pH 7.4, the induction of β-structures was evident on the C-native segment when incubated with PC vesicles, both SUVs and LUVs (Figure 3C,D). Interestingly, this phenomenon did not occur when the F23R variant was used (Figure 3E,F). Since several studies have reported that cholesterol (Chol) functions as a trigger factor of amyloid formation [33,34], unilamellar PC/Chol vesicles (prepared at a 3/2 molar ratio) were evaluated. Despite the increase of PC/Chol SUVs concentration (0–180 µM), our data suggest that PC/Chol vesicles do not promote β-sheet formation on the F_23_R variant (Figure 3G,H), whereas the I_26_A variant shows the formation of β-sheet structures (Figure 3G,H).

**Figure 4:**
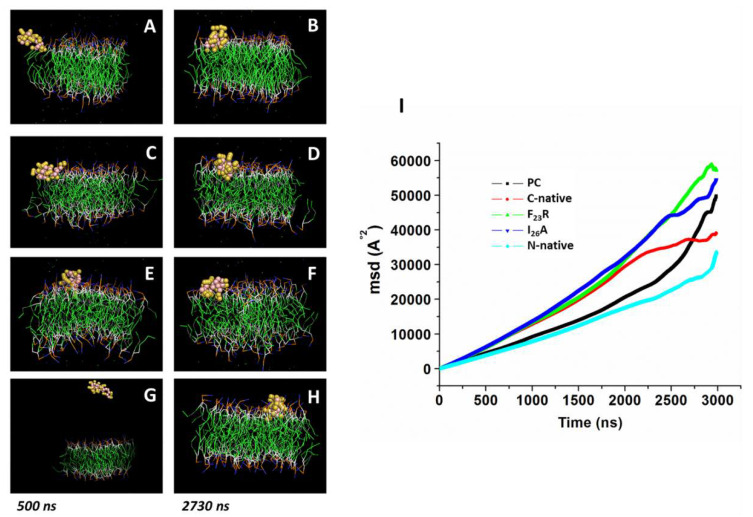
Displacement of peptides derived from IAPP on the *z*-axis of PC-bilayers (mean square deviation, MSD). Representative snapshots of PC bilayers associated with the C-native fragment of IAPP at 500 ns (**A**) and 2730 ns (**B**), F_23_R (**C**,**D**), I_26_A (**E**,**F**), and the N-native fragment (**G**,**H**). (**I**) Behavior of mean square deviation (Å^2^) through a 3000 ns simulation employing coarse grain methodology.

In a complementary way, we performed molecular dynamic simulations according to our experimental conditions using PC molecules. Simulations were conducted in the presence of modeled peptides (C-native segment, variants F_23_R, I_26_A, and N-native segment) and lipid bilayers with no peptides used as control. We ran simulations during 3000 ns in all systems, and the peptide molecule was situated at 30 Å from the top of the membrane. Although the three peptides derived from the hIAPP C-terminal domain (C-native, F_23_R, and I_26_A) adsorb at the membrane surface at less than 500 ns (Figure 4A–F), we found a slightly lower displacement for the C-native segment concerning the F_23_R and I_26_A during the evaluation of the displacement of phospholipids and peptide MSD (mean square deviation) on the *z*-axis, suggesting that interactions between C-native/lipids generate a movement restriction (Figure 4I). This phenomenon could be related to peptide interactions on the hydrophilic/hydrophobic interface of membranes. Moreover, to robust our experimental system and in order to discard false-positive bias, complementary MSD analysis (Figure S3) was performed through 100 consecutive short simulations (30 ns) for each system. According to the obtained results, the displacement of phospholipids and peptides derived from IAPP on the *z*-axis, follows the same behavior described in Figure 4I (Figure S3).

Several reports indicate that the region that initially interacts with cell membranes is the N-terminal of hIAPP [35]; our simulations suggest that the adsorption phenomenon of the N-native segment at the lipid interface (Figure 4G,H) presents an even greater restriction of movement with respect to peptides derived from the C-domain segment (Figure 4I).

### 3.3. Structure Modulation Dependent on Negative Electrostatic Surface in hIAPP Peptides

**Figure 5:**
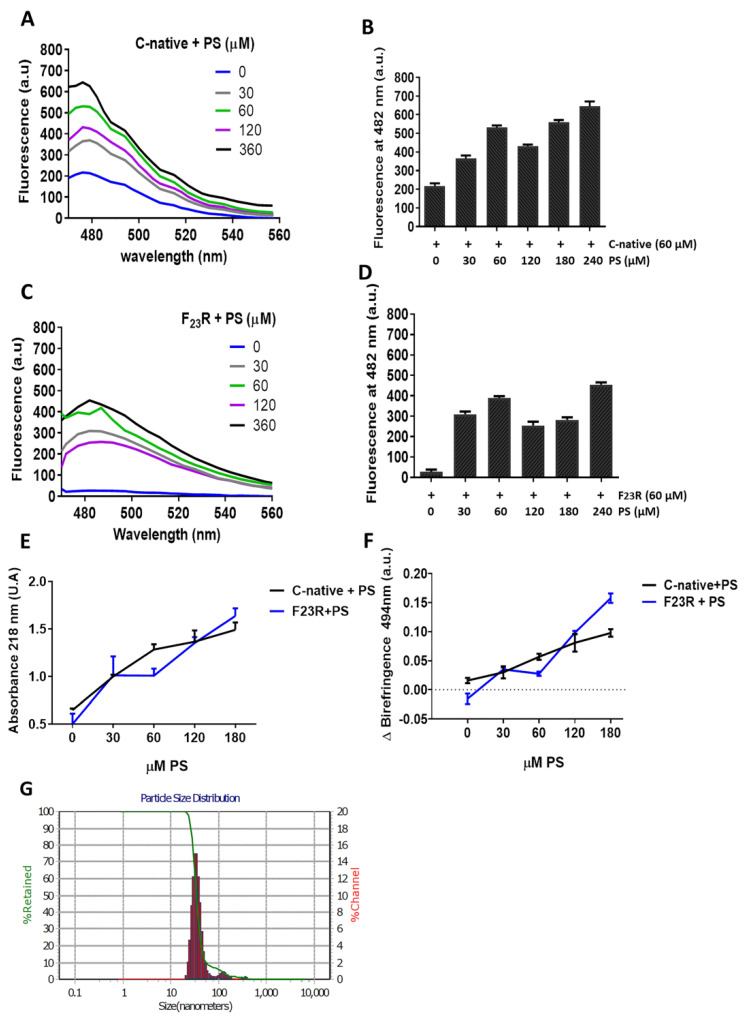
PS-SUVs promote the formation of β-sheet structures on the C-terminal-derived peptides of IAPP. (**A**) Interaction of the C-native segment at increasing concentrations of PS evaluated by ThT-fluorescence assays. (**B**) Same experimentation as in A, ThT-fluorescence at 482 nm. (**C**) Fluorescence emission spectra obtained through ThT assays on the F_23_R variant under increasing concentrations of PS. (**D**) Same experimentation as in (**C**), ThT-fluorescence at 482 nm. (**E**) Evaluation of the PS-effect on the secondary structure of peptides through peptide-bond absorbance at 218 nm, and by Congo red birefringence at 494 nm (**F**). (**G**) Dispersion of the size of PS-SUVs used in this experimentation by DLS.

Insulin secretory granules are derived from the ER membrane, contain high levels of PS and phosphatidylinositol in comparison to the plasmatic membrane [16], and show an important dynamic behavior [17]. Therefore, in order to evaluate the impact of anionic electrostatic charge, PS and POPG vesicles were evaluated (Figure 5). In a first assay, our results suggest a modulation by PS-SUVs on the C-native segment, wherein a significant increase in ThT-fluorescence values dependent on PS concentration was identified (Figure 5A,B). The F_23_R variant showed the same β-sheet formation from the lowest PS concentration (30 μM) (Figure 5C,D), a result that contrasts with the effect found when zwitterionic PC and PC/Chol vesicles are used. In complementary experimentation, the same tendency was registered through peptide bond spectrophotometry and Congo red assays (Figure 5E,F). Results confirm the propensity of the F_23_R variant to show β-sheet transitions induced by PS-SUVs, supporting the fact that the fibrillation process begins when monomers change to oligomers and that, in general, the formation of mature fibrils follow a sigmoidal behavior [36].

Moreover, although we registered the effect of PS-SUVs on β-sheet conformational transitions in hIAPP-derived peptides, we performed several efforts to obtain a homogenate LUV-preparation, however, considering the intrinsic PS-property to form SUVs, we designed a protocol based in freezing/thawing and a slightly sonication process (Materials and Methods). Then, we obtained a mixture of SUVs and LUVs. Results suggest the presence of the same β-sheet transitions registered in the C-native and F_23_R variant under treatment with SUVs/LUVs mixtures (Figure S4).

**Figure 6:**
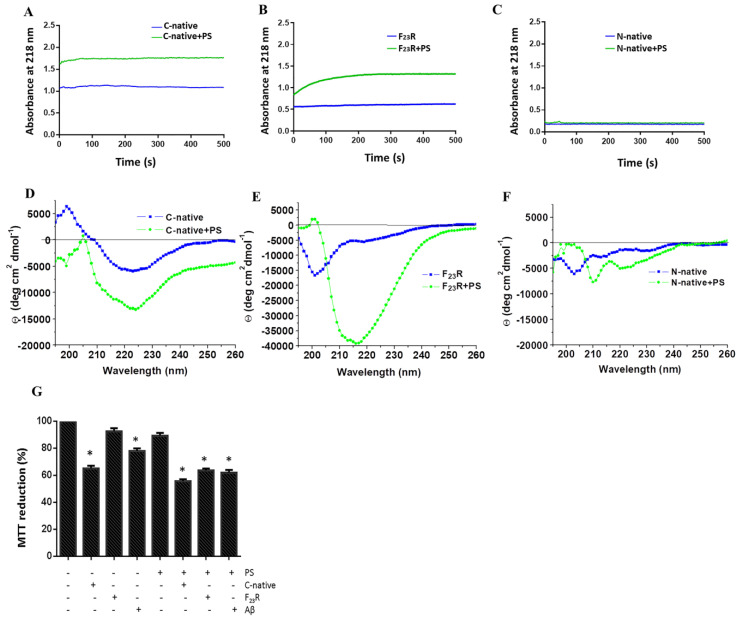
The anionic lipid surface as the critical factor for β-sheet aggregation on C-terminal peptides. Effect of PS on nucleation of the C-native segment (**A**), F_23_R variant (**B**), and the N-native segment (**C**). Same experimentation as in A, B, and C, the effect of PS on the secondary structure of the C-native segment (**D**), F_23_R variant (**E**), and N-native segment (**F**), evaluated by CD. (**G**) Cell viability evaluation on RIN-m5F cells treated under different stimuli of peptides/PS vesicles (7.5/15 μM). Data are expressed as mean ± SD (*n* = 6) * *p* < 0.005.

To perform an approximation to the nucleation phenomenon of hIAPP-derived peptides at a 1/2 peptide/PS ratio, peptide bond conformation was monitored along with time (Figure 6A). Data suggest that the incubation with PS-SUVs triggers a conformational transition to β-sheet formation, shortening the lag phase [37,38]. Variant F_23_R shows the same tendency under the interaction with anionic PS-vesicles (Figure 6B). Complementary results obtained through CD confirmed the same phenomenon with the C-native segment and the F_23_R variant (Figure 6D,E). Once the lag phase is surpassed, the oligomeric structures establish interchain electrostatic interactions to evolve into more ordered structures and amyloid fibrils [36]. In this case, this phenomenon was critical for the F_23_R variant, showing an eightfold increase in the CD signal at 222 nm, corresponding to the formation of β-sheet structures. In this sense, arginine and cationic residues have been described to interact with PS membranes due to high affinity of the side chains for anionic lipids [39]. These β-sheet transitions were not evident in the N-domain segment despite of positive charge content (Figure 6C) at neutral pH (Table 1); however, according to CD spectra, this interaction facilitates α-helix formation (Figure 6F). Furthermore, the structural transitions promoted by PS (Figure 6A,B) were not evidenced when vesicles composed of phosphatidyl-ethanolamine in both the C-native and N-native segments were evaluated (Figure S5). Then, the cationic lipid surface is not a critical factor for β-sheet aggregation on hIAPP segments.

Strikingly, on the other hand, variants F_23_R and I_26_A showed aggregation upon their interaction with the anionic lipid system (PS), as evidenced by CD spectra (Figure 6E). These results appear to be contradictory considering the AGGRESCAN values (Na4vSS), −5.3 for F_23_R and −8.2 for I_26_A (Table 1), which predict that the formation of β-structures should be inhibited, in association with reports suggesting that membranes of β-cells are composed of anionic lipids (2.5–13%) [40]. Taking into account this rather unexpected outcome, cell viability assays were performed in a model of Langerhans islet β-cells (RIN-m5F cells) to assess the effect of peptide/PS vesicles mixtures at the cellular level. Results pointed out that F_23_R/PS mixtures decreased cell viability compared to controls. Likewise, when the anionic vesicles are present, the C-native segment and the F_23_R fragment showed cytotoxicity properties (Figure 6G). In this experimental design, the Aβ peptide was used as a control.

**Figure 7:**
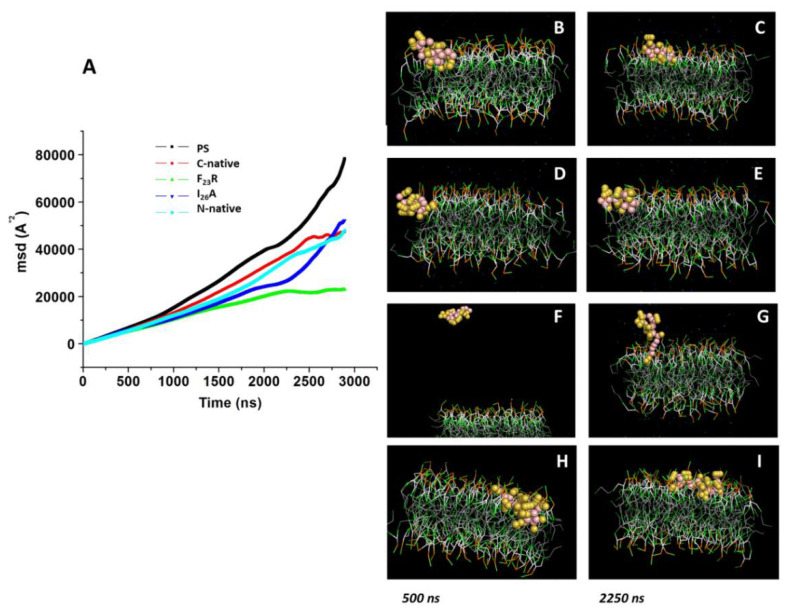
Displacement of peptides derived from the IAPP C-terminal on the *z*-axis of PS-bilayer. (**A**) MDS values on PS bilayer under the incubation of peptides-derived of IAPP through 3000 ns of simulation. Representative snapshots of the PS model with the C-native segment at 500 ns (**B**) and 2250 ns (**C**). Snapshots of F_23_R (**D**,**E**), I_26_A (**F**,**G**), and the N-native segment (**H**,**I**) under the same conditions.

As a complementary test, we performed dynamic simulations of IAPP-derived peptides in the presence of a preassembled PS bilayer during 3000 ns, wherein the peptide molecule was situated at 30 Å above the top of the membrane in all systems (Figure 7). In the evaluation of the displacement of the interacting species (PS and peptides), we found a considerable diminution in MSD for the F_23_R variant with respect to the C-terminal segment and I_26_A (Figure 7A), which is possibly associated with an electrostatic factor. Indeed, in the analysis of the trend line, the F_23_R slope showed the lowest value compared to results obtained with other peptides (data not showed). In the same way that of the PC system, a complementary MSD analysis through 100 consecutive short simulations (30 ns) for each PS-system was performed, and results obtained from these short simulations follow the same tendency (Figure S6). Moreover, Aβ-peptide was incorporated as a control. Importantly, through the simulation time, the collapse of the simulations was not registered. Thus, interactions between the F_23_R and lipids generate a restriction in movement modifying the lipid packing and show the F_23_R peptide situated deeper within the lipid bilayer (Figure 7D,E). According to our CD experimentation, this is possibly a critical condition for the propensity modulation towards the formation of β-sheet structures.

To determine whether the PS anionic environment is the factor that leads to the formation of β-structures, other anionic lipid vesicles were evaluated. To this end, vesicles of PC and anionic-POPG were prepared at a 3 to 1 molar ratio, for which, the characterization of the peptide bond at 218 nm showed for the C-native segment, F_23_R, and I_26_A variants, an increase in peptide bond absorbance, demonstrating a phosphatidyl-glycerol (POPG)-modulation dependent response (Figure S7A). In the same way, the three fragments, C-native segment, F_23_R, and I_26_A, incubated with POPG enhanced cytotoxicity on β-cells (Figure S7B). Likewise, a control with Aβ-peptide was included. To extend this characterization using peptide-bond spectroscopy, anionic SUVs were evaluated by increasing the molar concentration of POPG (0–100%). A strong modulation of POPG on the β-structure of the C-native fragment and both F_23_R and I_26_A was observed (Figure S7C). Therefore, it seems that anionic electrostatic charges prove to play an important role in amyloidogenesis, as further demonstrated by the evaluation of a representative Aβ-peptide (Figure S7C).

Although the structure of IAPP could change according to the physicochemical properties of lipids [7], in an attempt to characterize the role of lipid surfaces on the conformational transitions of β-sheets, we evaluated the influence of solvation determined by the presence of a free hydroxyl group in position 2 of phospholipid heads. Our results indicate that treatment with lysophosphatidic acid (LPA) micelles under a neutral pH promotes conformational transitions towards β-sheet structures only in the C-native segment and F_23_R variant (Figure S8). For the case of the N-native segment, a spectrum corresponding to an α-helix signal was registered, reflecting the avoidance of β-sheet structure formation. These results appear to confirm that β-structure modulation is localized at the C-native domain of IAPP (Figure S8). Results that are in concordance with the proposal that amyloidogenicity and cytotoxicity are induced by two different regions of the hIAPP sequence [41]. Likewise, these findings are in agreement with previous results from our laboratory where we described that incubation with LPA promotes conformational transitions in the C-terminal domain of cholesteryl ester transfer protein (CETP) [10].

## 4. Discussion

During the course of the present investigation, employing a biomimetic approach, we have been able to develop and evaluate new variants of IAPP with the property to show fewer propensities to form β-sheet structures. New sequences such as F_23_R have shown to increase the stability of the C-native segment of IAPP, with an important involvement of the anionic nature lipids. This behavior can be associated at pH 7.4 with charged lipids that contributed to structural changes towards the β-sheet formation, whereas the I_26_A variant shows a neutral charge and an isoelectric point similar to the C-native segment, promoting a strong interaction with anionic phospholipids. Molecular dynamics showing an absorption phenomenon of F_23_R at the surface of lipid bilayer indicate the possibility for the formation of peptide-PS aggregates that, by means of lipotoxicity, could have contributed to the observed cytotoxicity (Figure 6G). Further experiments are nowadays in progress in our laboratories to confirm this assumption (data to be published).

The content of anionic phospholipids in β-cell membranes is reported to range from 2.5% to 13.2%, a proportion mainly situated in the inner leaflet of the membrane [38], proportion much similar as found in insulin secretory granules where a higher content of PS and phosphatidylinositol has been described [17,42], which are derived from ER membranes during insulin maturation. This contributes to the possibility that hIAPP aggregation could be increased at the intracellular space, especially during the process of maturation of insulin secretory granules. The phenomenon of membrane asymmetry that could enhance hIAPP amyloid formation and membrane damage in vivo [43] could modify the PS-biodisponibility and, therefore, triggers the β-sheets formation.

**Figure 8:**
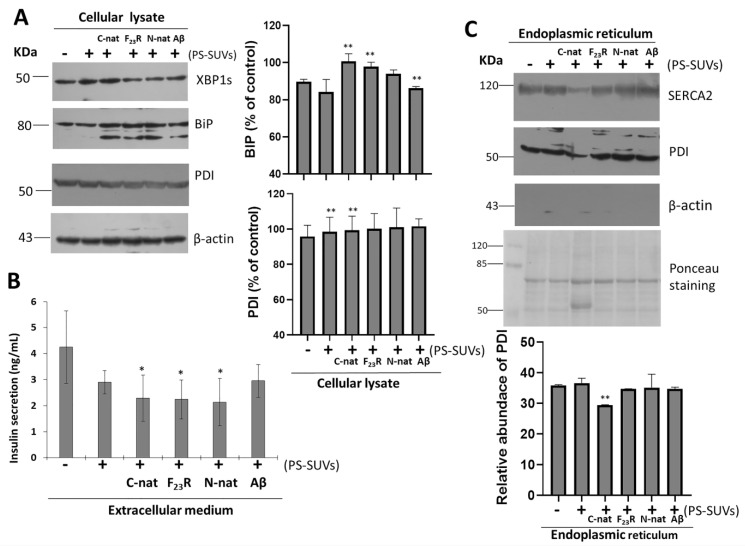
Unfolded protein response is activated under the treatment of hIAPP-derived peptides/PS vesicles mixtures. (**A**) Effect of hIAPP-derived peptides/PS on the expression of XBP1s and binding immunoglobulin protein (BiP) targets of unfolded protein response (UPR), as well as protein disulfide isomerases (PDI) expression on complete cell lysates. Densitometry analysis of (BiP) and PDI immunoblots, results are reported as means ± SD and expressed as % of control, ** *p* < 0.05. Aβ-peptide was used as a control. β-actin was used as a loading control. (**B**) Insulin concentrations (ng/mL) in extracellular media, * *p* < 0.1. (**C**) Under the same experimental conditions, the ER was purified and the expression of sarco/endoplasmic reticulum Ca^2+^-ATPase-2 (SERCA2), PDI, and β-actin were characterized. Polyvinylidene difluoride (PVDF) membranes were stained with Ponceau. Densitometry analysis of PDI immunoblots; results are reported as means ± SD and expressed as relative abundance; ** *p* < 0.05.

Therefore, considering the critical role of PS in the promotion of β-sheet conformational transitions on hIAPP-derived peptides, the impact of phospholipid negative-electrostatic charge on UPR regulation was characterized. In this sense, PS-SUVs with the hIAPP-derived peptides under a 1 to 2 peptide/PS ratio were incubated on β-cell cultures for 20 h. In the first case, critical UPR-targets were evaluated under the peptide/PS treatments in complete cellular lysates (Figure 8A) results suggest the slight activation of XBPIs, a transcription factor of the activation of IRE1-arm of UPR. Likewise, an increase in the expression of chaperone BiP under peptides/PS treatments was registered, and this phenomenon was more evident under the C-native treatment. Possibly, these modifications could be partly related to a cellular compensatory response aimed to maintain protein homeostasis in ER (Figure 8A), as well as critical functions in the physiology of β-cells. Importantly, when insulin concentrations were evaluated in extracellular media, we found a diminution of the insulin levels upon treatment with the peptide/PS mixtures (Figure 8B).

PDI is a chaperone that regulates folding of proinsulin, participates in disulfide bond formation, and maintains ER redox homeostasis [44]. In our conditions, when total β-cell lysates were evaluated under peptide/PS treatments, we did not find changes in PDI expression (Figure 8A). Constituting a chaperone-protein with critical functions, high expression levels of PDI have been found in the ER lumen, to a lower extend in the cytosol, and also in different cellular membranes. [45]. In addition, we have also detected PDI in the extracellular medium of β-cells treated with peptide/PS mixtures (data not shown). However, more experimental evidence is required to establish a mechanistic proposal.

To dissect the role of PDI, we performed the isolation of ER of β-cells cultures, evaluating sarco/endoplasmic reticulum Ca^2+^-ATPase-2 (SERCA2) and β-actin as controls of ER isolations. Results indicated the isolation of pure ER-fractions (Figure S9). Then, under the same treatments of hIAPP-peptides/PS-SUVs, we characterized the expression of PDI. We found that the PDI levels diminished upon treatment with C-native/PS, as well as the levels of SERCA2 resident of ER (Figure 8C). Results suggest that the affectation of SERCA2 and PDI under C-native/PS treatment might be related to both their ER localization and the activation of UPR, affecting insulin secretion.

**Figure 9:**
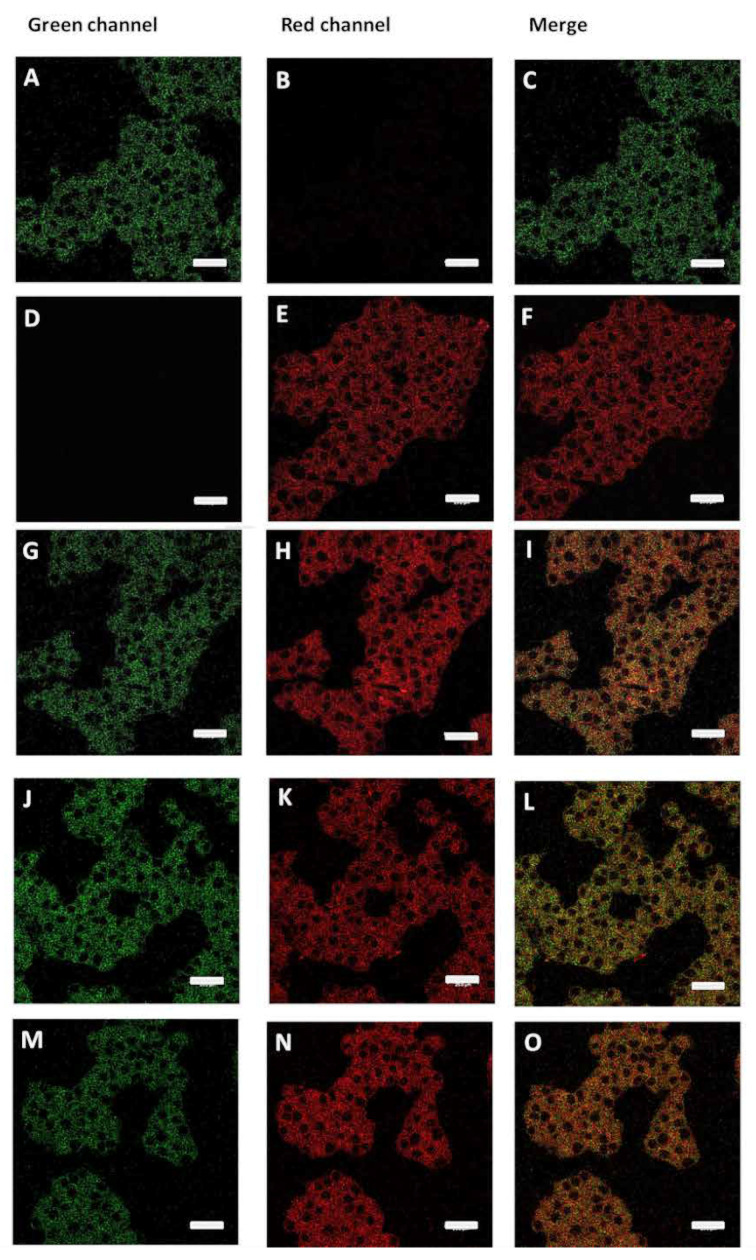
BODIPY-Leu/PS vesicles (green) colocalize with ER-tracker (red). (**A**–**C**) Langerhans islet β-cells (RIN-m5F cells) treated with BODIPY-Leu/PS vesicles. (**D**–**F**) Cells treated with ER-tracker. (**G**–**I**) Cells treated jointly with BODIPY-Leu/PS vesicles and ER-tracker. (**J**–**L**) Effect of C-native treatment on localization of BODIPY-Leu/PS vesicles and ER-tracker. (**M**–**O**) Effect of F_23_R peptide treatment on localization of BODIPY-Leu/PS vesicles and ER-tracker. Scale bars correspond to 25 µm.

ER-lumen and the function of chaperones BiP and PDI are critical during proinsulin folding. Considering our results, and in an attempt to characterize the effect of the PS vesicles and possibly trace their cellular localization, confocal microscopy experiments were carried out. To this end, we prepared PS and peptide/PS vesicles (30 µM) tagged with the green fluorescent probe BODIPY-Leu [46] (6 µM) (referred to as BODIPY-Leu/PS and BODIPY-Leu/peptides/PS vesicles, respectively), with which RIN-m5F cells were treated. In the first instance, our results demonstrated that BODIPY-Leu/PS vesicles are internalized in RIN-m5F cells (Figure 9A–C, stained in green). Then, we used ER-tracker (red staining) for characterize the colocalizing in ER sites (Figure 9D–I). Results suggest that the system is located in the ER. Importantly, when we evaluated the localization of the BODIPY-Leu/PS vesicles incubated with hIAPP-derived peptides (C-native and F_23_R variant) under a molar relationship (1/2; peptide/vesicle), the BODIPY-Leu/PS signal was localized under C-native treatment in ER sites (Figure 9J–L), however, the signal diminished slightly under F_23_R treatment (Figure 9M–O). In an important way, C-native/PS treatment promoted the higher levels of cytotoxicity in the studied cells (Figure 6G), affecting insulin secretion (Figure 8B). Moreover, these phenomena coincide with UPR activation and affectation of localization of PDI and SERCA2 in the ER (Figure 8C). Therefore, it appears that the localization of peptide/PS is a critical condition to induce the alterations in homeostasis of the ER. This phenomenon was evidenced after treatment with the C-native/PS system, whereupon the higher fluorescence signal of the BODIPY-Leu/PS vesicles along the ER very likely corresponds with the alterations in localization of PDI, SERCA2, and the insulin secretion. This phenomenon was not evident upon treatment with the F_23_R variant.

Therefore, our results suggest that the lipid-anionic electrostatic charge is a critical condition that could modulate the UPR pathway and the conformational transition of IAPP-derived peptides. Thus, a negative electrostatic-charge environment could be critical in insulin secretion as well. Interestingly, the content of anionic lipids of β-cells and insulin secretory granules has been related to an altered glucose-stimulated insulin exocytosis [17,43]. Therefore, a condition of metabolic overload also has been associated with the biodisponibility of fatty acids [22], very likely contributing to the deleterious phenomenon correlated with the concentration of anionic phospholipids, promoting misfolded transitions on hIAPP. Having in mind this phenomenon, our group has generated new materials of polymeric films of polyvinyl dimethylazlactone (PVDMA) and polyethylene imine (PEI) to evaluate the effect of fatty acids on β-cell membranes [47] and diverse critical physiological functions.

In a complementary way, current results of our laboratory suggest that the induction of oligomers at the C-native domain of IAPP accelerates β-sheet formation when treated with oleic acid/PC vesicles. By contrast, when palmitic acid/PC vesicles are used, this result is not found (data not shown). Then, considering that unsaturation and shorter fatty acids of phospholipids facilitate the curvature and fluidity of membranes favoring their fusion [17], although increasing the risk of aggregation, there is a subtle regulation in the conservation of the structure of the hIAPP. Moreover, a report reveals synergic implications of free fatty acids and hIAPP in ER stress and apoptosis of islet β-cells [48]. In this context, we have documented the role of metabolic overload by saturated fatty acids on proteostasis and its impact on insulin secretion, specifically the dysregulation of targets that control intracellular calcium homeostasis [22], as also documented in this report for SERCA2.

Recently, following peptidomimetic design strategies, research has been developed to find a way to inhibit the formation of β-sheet structures in segments of an important series of polypeptides and proteins as a therapeutic way to fight amyloid disease. There is still a field of action in amyloidogenesis design. Although prolines residues in rIAPP promote disordered structures, results of coincubation of rIAPP and hIAPP suggest that rat amylin does not block β-sheet and also forms its own β-sheet, most probably on the outside of the human fibrils [49], revealing the complex behavior in the development of an amyloid fibril inhibitor. We and other authors have documented that it is critical to consider the impact of lipid environment. In the light of our results, the F_23_R variant of IAPP showed a low propensity to form β-sheet structures even under the effect of zwitterionic lipids. However, anionic charge of lipid vesicles and degree of solvation were factors for the modulation of β-sheet formation of the F_23_R and I_26_A variants, as well as in the C-native segment of IAPP, all associated to the cytotoxicity phenomena of β-cells. In conclusion, our results show the potential implications of modulating the structure and stability of IAPP for the design of analog therapeutics based on peptides and proteins.

## Figures and Tables

**Table 1:** Physicochemical parameters of evaluated peptides.

ID	Sequence	AGGRESCAN Value ^1^	Hydropathy (kcal/mol)	Charge pH 7.4	Hydrophobicity (kcal/mol)	pI	µH kcal/mol
C-native	^23^FGAILSSTNGSNTY^37^	6.6	0.28	0	0.29	5.92	0.18
F_23_R variant	^23^RGAILSSTNGSNTY^37^	−0.3	−0.21	1	0.21	9.84	0.04
I_26_A variant	^23^FGAALSSTNGSNTY^37^	−8.2	0.1	0	0.24	5.92	0.13
N-native	^1^KCNTATCATQRLANFLVHSS^20^	−6.3	−0.04	3	−0.02	9.14	0.27

^1^ Negative values are associated with a low propensity to form amyloid fibrils, whereas positive ones are associated with higher propensity. Red identifies the residue variants.

## Supplementary Materials

The following are available online at https://www.mdpi.com/2218-273X/10/9/1201/s1, Figure S1. Incubation with SUVs composed of phosphatidylcholine does not induce conformational transitions on the N-native segment (^1^KCNTATCATQRLANFLVHSS^20^) of hIAPP. Figure S2. Effect of PC-LUVs on the secondary structure of peptides derived from hIAPP. Figure S3. Displacement of peptides on the *z*-axis of PC-bilayers (MSD) obtained by short simulations. Figure S4. Mixtures of LUVs/SUVs composed of PS facilitate the formation of β-sheet structures. Figure S5. The cationic lipid surface is not a critical factor for β-sheet aggregation on hIAPP segments. Figure S6. Displacement of peptides on the *z*-axis of PS-bilayers (MSD) obtained by short simulations. Figure S7. Effect of SUVs composed of 1-palmitoyl-2-oleoyl-sn-glycerol (POPG) on the structure of IAPP variants. Figure S8. Effect of lysophosphatidic acid incubation on the secondary structure of C-native (**A**), F_23_R variant (**B**), and N-native (**C**). Figure S9. Characterization of several fractions obtained during endoplasmic reticulum (ER) isolation.

## References

[1] Koda J.E., Fineman M., Rink T.J., Dailey G.E., Muchmore D.B., Linarelli L.G. (1992). Amylin concentrations and glucose control. Lancet.

[2] Percy A.J., Trainor D.A., Rittenhouse J., Phelps J., Koda J.E. (1996). Development of sensitive immunoassays to detect amylin and amylin-like peptides in unextracted plasma. Clin. Chem..

[3] Novials A., Sarri Y., Casamitjana R., Rivera F., Gomis R. (1993). Regulation of islet amyloid polypeptide in human pancreatic islets. Diabetes.

[4] Xu W., Jiang P., Mu Y. (2009). Conformation preorganization: Effects of S20G mutation on the structure of human islet amyloid polypeptide segment. J. Phys. Chem. B.

[5] Poa N.R., Cooper G.J., Edgar P.F. (2003). Amylin gene promoter mutations predispose to Type 2 diabetes in New Zealand Maori. Diabetologia.

[6] Berhanu W.M., Hansmann U.H. (2014). Inter-species cross-seeding: Stability and assembly of rat-human amylin aggregates. PLoS ONE.

[7] Pulido-Capiz A., Diaz-Molina R., Martinez-Navarro I., Guevara-Olaya L.A., Casanueva-Perez E., Mas-Oliva J., Rivero I.A., Garcia-Gonzalez V. (2018). Modulation of Amyloidogenesis Controlled by the C-Terminal Domain of Islet Amyloid Polypeptide Shows New Functions on Hepatocyte Cholesterol Metabolism. Front. Endocrinol..

[8] Garcia-Gonzalez V., Gutierrez-Quintanar N., Mas-Oliva J. (2015). The C-terminal Domain Supports a Novel Function for CETPI as a New Plasma Lipopolysaccharide-Binding Protein. Sci. Rep..

[9] Garcia-Gonzalez V., Gutierrez-Quintanar N., Mendoza-Espinosa P., Brocos P., Pineiro A., Mas-Oliva J. (2014). Key structural arrangements at the C-terminus domain of CETP suggest a potential mechanism for lipid-transfer activity. J. Struct. Biol..

[10] Garcia-Gonzalez V., Mas-Oliva J. (2013). Amyloid fibril formation of peptides derived from the C-terminus of CETP modulated by lipids. Biochem. Biophys. Res. Commun..

[11] Diaz-Villanueva J.F., Diaz-Molina R., Garcia-Gonzalez V. (2015). Protein Folding and Mechanisms of Proteostasis. Int. J. Mol. Sci..

[12] Garcia-Gonzalez V., Mas-Oliva J. (2011). Amyloidogenic properties of a D/N mutated 12 amino acid fragment of the C-terminal domain of the Cholesteryl-Ester Transfer Protein (CETP). Int. J. Mol. Sci..

[13] Mendoza-Espinosa P., Garcia-Gonzalez V., Moreno A., Castillo R., Mas-Oliva J. (2009). Disorder-to-order conformational transitions in protein structure and its relationship to disease. Mol. Cell. Biochem..

[14] Knight J.D., Hebda J.A., Miranker A.D. (2006). Conserved and cooperative assembly of membrane-bound alpha-helical states of islet amyloid polypeptide. Biochemistry.

[15] Jayasinghe S.A., Langen R. (2005). Lipid membranes modulate the structure of islet amyloid polypeptide. Biochemistry.

[16] Suckale J., Solimena M. (2010). The insulin secretory granule as a signaling hub. Trends Endocrinol. Metab..

[17] MacDonald M.J., Ade L., Ntambi J.M., Ansari I.U., Stoker S.W. (2015). Characterization of phospholipids in insulin secretory granules and mitochondria in pancreatic beta cells and their changes with glucose stimulation. J. Biol. Chem..

[18] Saito Michiko S.Y. (2019). ER Stress, Secretory Granule Biogenesis, and Insulin. Ultimate Guide to Insulin.

[19] Schuck S., Prinz W.A., Thorn K.S., Voss C., Walter P. (2009). Membrane expansion alleviates endoplasmic reticulum stress independently of the unfolded protein response. J. Cell. Biol..

[20] Meyerovich K., Ortis F., Allagnat F., Cardozo A.K. (2016). Endoplasmic reticulum stress and the unfolded protein response in pancreatic islet inflammation. J. Mol. Endocrinol..

[21] Galindo-Hernandez O., Cordova-Guerrero I., Diaz-Rubio L.J., Pulido-Capiz A., Diaz-Villanueva J.F., Castaneda-Sanchez C.Y., Serafin-Higuera N., Garcia-Gonzalez V. (2019). Protein translation associated to PERK arm is a new target for regulation of metainflammation: A connection with hepatocyte cholesterol. J. Cell. Biochem..

[22] Acosta-Montano P., Rodriguez-Velazquez E., Ibarra-Lopez E., Frayde-Gomez H., Mas-Oliva J., Delgado-Coello B., Rivero I.A., Alatorre-Meda M., Aguilera J., Guevara-Olaya L. (2019). Fatty Acid and Lipopolysaccharide Effect on Beta Cells Proteostasis and its Impact on Insulin Secretion. Cells.

[23] Conchillo-Sole O., de Groot N.S., Aviles F.X., Vendrell J., Daura X., Ventura S. (2007). AGGRESCAN: A server for the prediction and evaluation of "hot spots" of aggregation in polypeptides. BMC Bioinform..

[24] Szoka F., Papahadjopoulos D. (1980). Comparative properties and methods of preparation of lipid vesicles (liposomes). Annu. Rev. Biophys. Bioeng..

[25] Knapp B., Ospina L., Deane C.M. (2018). Avoiding False Positive Conclusions in Molecular Simulation: The Importance of Replicas. J. Chem. Theory Comput..

[26] Losasso V., Pietropaolo A., Zannoni C., Gustincich S., Carloni P. (2011). Structural role of compensatory amino acid replacements in the alpha-synuclein protein. Biochemistry.

[27] Prajapati P., Wang W.X., Nelson P.T., Springer J.E. (2020). Methodology for Subcellular Fractionation and MicroRNA Examination of Mitochondria, Mitochondria Associated ER Membrane (MAM), ER, and Cytosol from Human Brain. Methods Mol. Biol..

[28] Patil S.M., Alexandrescu A.T. (2015). Charge-Based Inhibitors of Amylin Fibrillization and Toxicity. J. Diabetes Res..

[29] Balbach J.J., Ishii Y., Antzutkin O.N., Leapman R.D., Rizzo N.W., Dyda F., Reed J., Tycko R. (2000). Amyloid fibril formation by A beta 16-22, a seven-residue fragment of the Alzheimer’s beta-amyloid peptide, and structural characterization by solid state NMR. Biochemistry.

[30] Smaoui M.R., Waldispuhl J. (2015). Complete characterization of the mutation landscape reveals the effect on amylin stability and amyloidogenicity. Proteins.

[31] Cho W.J., Jena B.P., Jeremic A.M. (2008). Nano-scale imaging and dynamics of amylin-membrane interactions and its implication in type II diabetes mellitus. Methods Cell. Biol..

[32] Khemtemourian L., Domenech E., Doux J.P., Koorengevel M.C., Killian J.A. (2011). Low pH acts as inhibitor of membrane damage induced by human islet amyloid polypeptide. J. Am. Chem. Soc..

[33] Cho W.J., Trikha S., Jeremic A.M. (2009). Cholesterol regulates assembly of human islet amyloid polypeptide on model membranes. J Mol Biol.

[34] Simons K., Toomre D. (2000). Lipid rafts and signal transduction. Nat. Rev. Mol. Cell. Biol..

[35] Skeby K.K., Andersen O.J., Pogorelov T.V., Tajkhorshid E., Schiott B. (2016). Conformational Dynamics of the Human Islet Amyloid Polypeptide in a Membrane Environment: Toward the Aggregation Prone Form. Biochemistry.

[36] DeToma A.S., Salamekh S., Ramamoorthy A., Lim M.H. (2012). Misfolded proteins in Alzheimer’s disease and type II diabetes. Chem. Soc. Rev..

[37] Kelly J.W. (2000). Mechanisms of amyloidogenesis. Nat. Struct. Biol..

[38] Xu W., Wei G., Su H., Nordenskiold L., Mu Y. (2011). Effects of cholesterol on pore formation in lipid bilayers induced by human islet amyloid polypeptide fragments: A coarse-grained molecular dynamics study. Phys. Rev. E.

[39] Vorobyov I., Allen T.W. (2011). On the role of anionic lipids in charged protein interactions with membranes. Biochim. Biophys. Acta.

[40] Platre M.P., Jaillais Y. (2017). Anionic lipids and the maintenance of membrane electrostatics in eukaryotes. Plant Signal. Behav..

[41] Martel A., Antony L., Gerelli Y., Porcar L., Fluitt A., Hoffmann K., Kiesel I., Vivaudou M., Fragneto G., de Pablo J.J. (2017). Membrane Permeation versus Amyloidogenicity: A Multitechnique Study of Islet Amyloid Polypeptide Interaction with Model Membranes. J. Am. Chem. Soc..

[42] Saitta F., Signorelli M., Fessas D. (2019). Dissecting the effects of free fatty acids on the thermodynamic stability of complex model membranes mimicking insulin secretory granules. Colloids Surf. B Biointerfaces.

[43] Zhang X., St Clair J.R., London E., Raleigh D.P. (2017). Islet Amyloid Polypeptide Membrane Interactions: Effects of Membrane Composition. Biochemistry.

[44] Jang I., Pottekat A., Poothong J., Yong J., Lagunas-Acosta J., Charbono A., Chen Z., Scheuner D.L., Liu M., Itkin-Ansari P. (2019). PDIA1/P4HB is required for efficient proinsulin maturation and ss cell health in response to diet induced obesity. eLife.

[45] Ali Khan H., Mutus B. (2014). Protein disulfide isomerase a multifunctional protein with multiple physiological roles. Front Chem..

[46] Avelino Jorge G.-G.V., Alatorre-Meda M., Rodríguez-Velázquez E., Rivero I.A. (2020). Synthesis of BODIPY-amino acids and the potential applications as specific dyes for the cytoplasm of Langerhans β-cells.

[47] Avila-Cossio M.E., Rivero I.A., Garcia-Gonzalez V., Alatorre-Meda M., Rodriguez-Velazquez E., Calva-Yanez J.C., Espinoza K.A., Pulido-Capiz A. (2020). Preparation of Polymeric Films of PVDMA-PEI Functionalized with Fatty Acids for Studying the Adherence and Proliferation of Langerhans beta-Cells. ACS Omega.

[48] Gao L.P., Chen H.C., Ma Z.L., Chen A.D., Du H.L., Yin J., Jing Y.H. (2020). Fibrillation of human islet amyloid polypeptide and its toxicity to pancreatic beta-cells under lipid environment. Biochim. Biophys. Acta Gen. Subj..

[49] Middleton C.T., Marek P., Cao P., Chiu C.C., Singh S., Woys A.M., de Pablo J.J., Raleigh D.P., Zanni M.T. (2012). Two-dimensional infrared spectroscopy reveals the complex behaviour of an amyloid fibril inhibitor. Nat. Chem..

