# Supplementary material for: Lipid Modulation in the Formation of β-Sheet Structures. Implications for De Novo Design of Human Islet Amyloid Polypeptide and the Impact on β-Cell Homeostasis
Source: Biomolecules. 2020 Aug 19;10(9):1201. doi: 10.3390/biom10091201 (PMC7563882; doi:10.3390/biom10091201)

Lipid modulation in the formation of β-sheet structures. Implications for *de novo* design of human islet amyloid polypeptide and the impact on β-cell homeostasis

Israel Martínez-Navarro [1,†], Raúl Díaz-Molina [1], Angel Pulido-Capiz [1,2,†], Jaime Mas-Oliva [3], Ismael Luna-Reyes [3], Eustolia Rodríguez-Velázquez [4,5], Ignacio A. Rivero [6], Marco A. Ramos-Ibarra [7], Manuel Alatorre-Meda [8] and Victor García-González [1,*]

Supplementary figures

Supplementary Figure 1. Incubation with SUVs composed of phosphatidylcholine (PC) does not induce conformational transitions on the N-native segment ([1]KCNTATCATQRLANFLVHSS[20]) of hIAPP. **A**) Evaluation by birefringence at 494 nm of N-native and C-native segments, under increasing concentrations of PC-vesicles. The effect of PC-vesicles (120 µM) on peptide bond absorbance at 218 nm in C-native (**B**) and N-native (**C**) fragments.

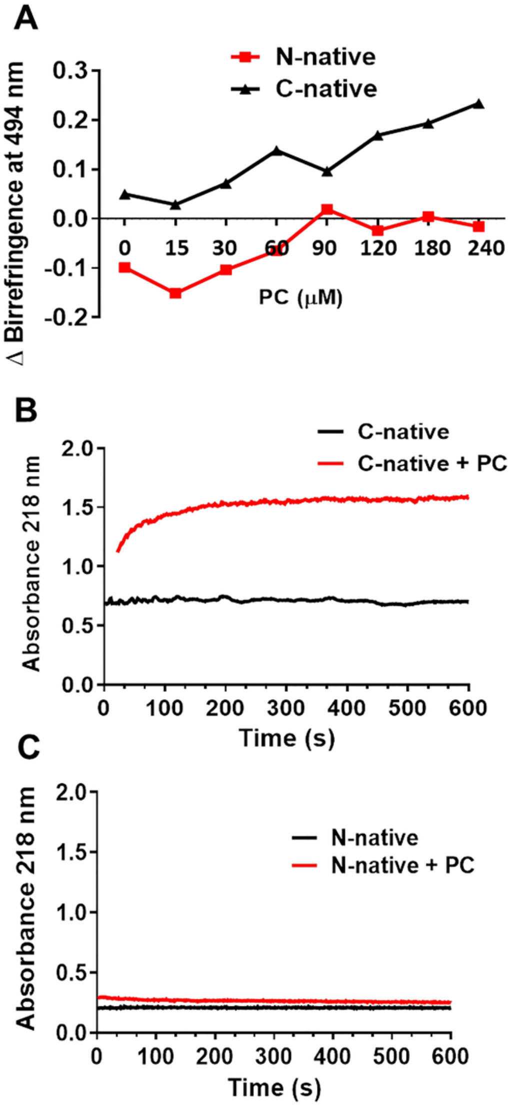

Supplementary Figure 2. Effect of PC-LUVs on the secondary structure of peptides derived from hIAPP. **A**) Peptide bond absorbance of the C-native segment and $F_{23}R$ under incubation with PC-LUVs. **B**) Under the same conditions, peptide characterization by birefringence with Congo-red assay. **C**) Representation of the LUVs size used in these assays, obtained through DLS experimentation.

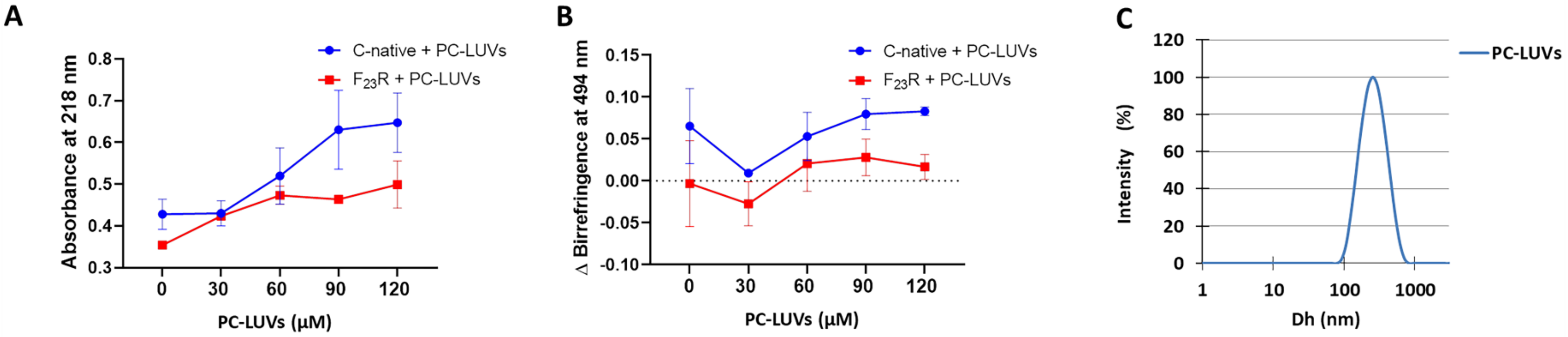

Supplementary Figure 3. Displacement of peptides on the *z*-axis of PC-bilayers (MSD) obtained by short simulations. Behavior of MSD (Å2) through 3000 ns simulation employing 100 consecutive simulations (30 ns) for each system. Using built-in functions of GROMACS to reach 3000 ns simulation time, all trajectories were joined, obtained from short simulations.

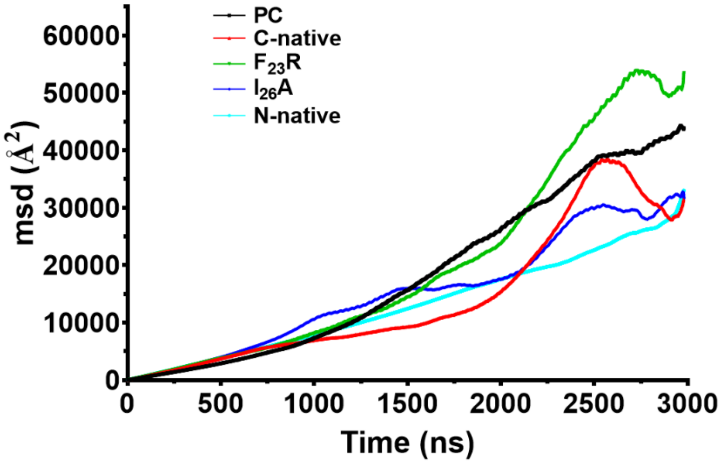

Supplementary Figure 4. Mixtures of LUVs/SUVs composed of PS facilitate the formation of β-sheet structures. Interaction of the C-native segment and $F_{23}R$ at increasing concentrations of PS mixtures evaluated through peptide-bond absorbance at 218 nm (**A**), and by Congo-red birefringence at 494 nm (**B**). **C**) Dispersion of the size of PS-vesicles used in this experimentation by DLS. Three peaks were registered, 1236 nm (volume 35.9 %), 357 nm (volume 42.8 %) and 84.2 nm (volume 21.3 %).

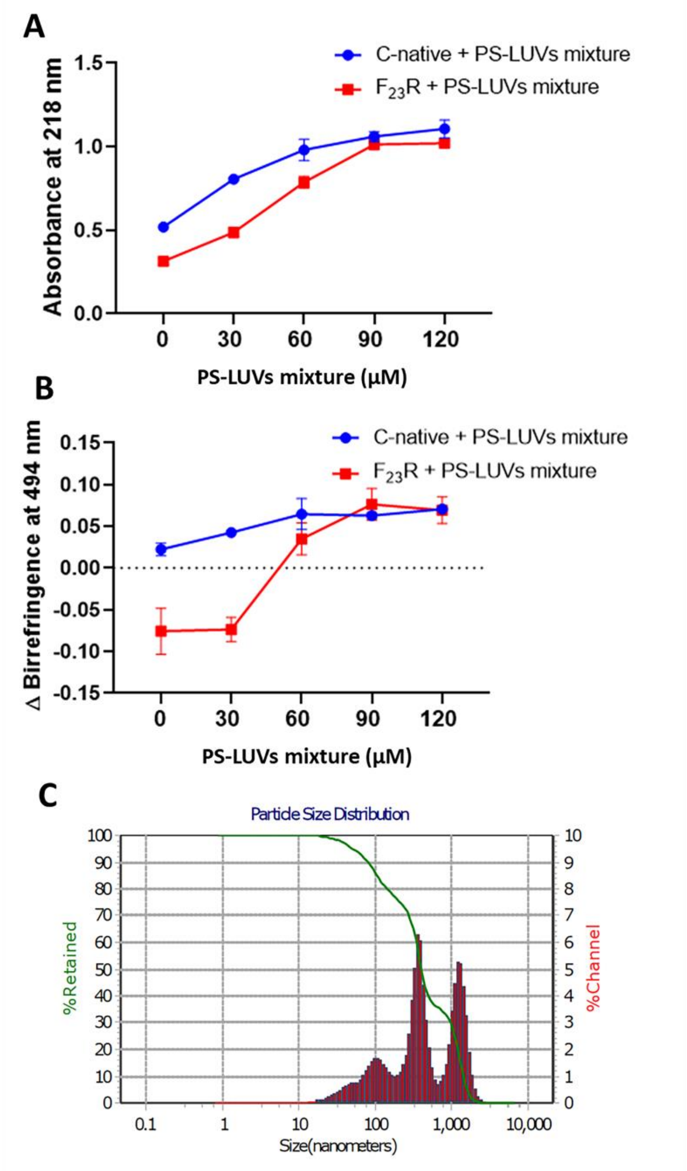

Supplementary Figure 5. The cationic lipid surface is not a critical factor for β-sheet aggregation on hIAPP segments. The effect of PE incubation on the nucleation of C-native (**A**), and N-native (**B**) evaluated by peptide bond absorbance.

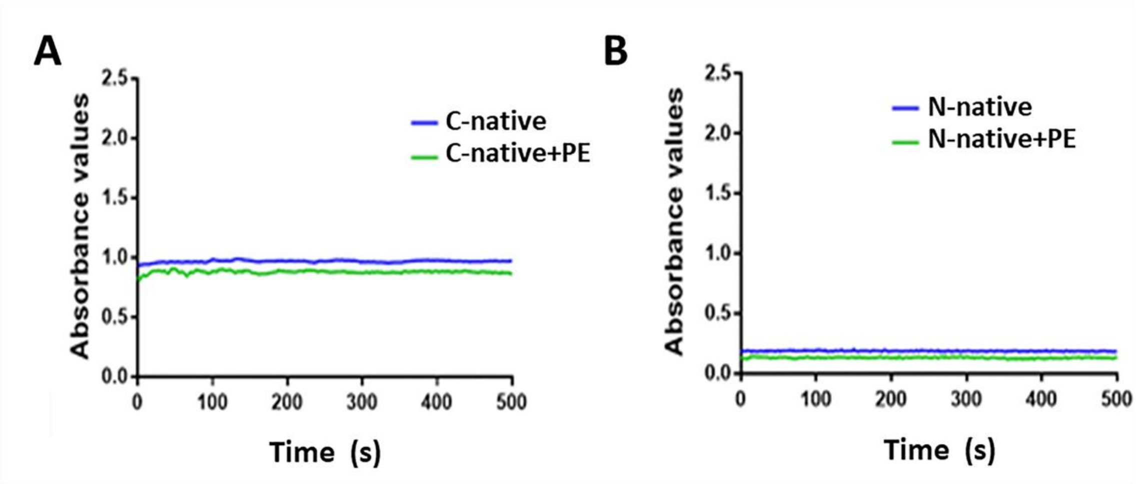

Supplementary Figure 6. Displacement of peptides on the *z*-axis of PS-bilayers (MSD) obtained by short simulations. Behavior of MSD (Å2) through 3000 ns simulation employing 100 consecutive simulations (30 ns) for each system.

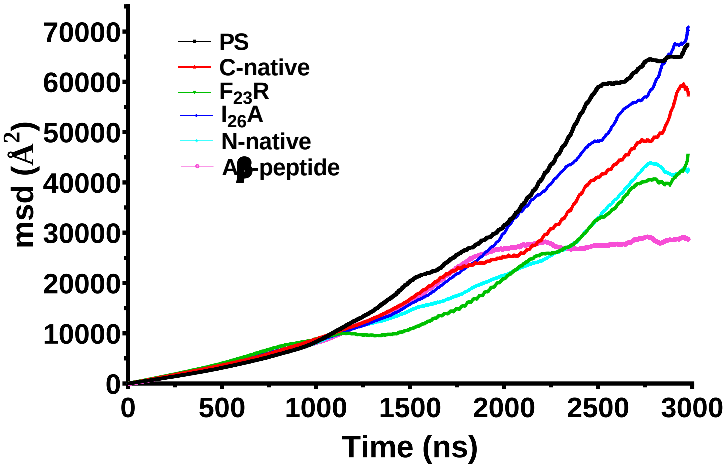

Supplementary Figure 7. Effect of SUVs composed of POPG on the structure of IAPP variants. **A**) Characterization of IAPP variants with POPG vesicles by peptide bond absorbance at 218 nm. **B**) Cell viability evaluation on RIN-m5F cells treated under different stimuli of peptides and POPG vesicles. **C**) Peptide-bond spectroscopy evaluated by several PC/POPG concentrations. Aβ peptide was used as a control. Mean values are presented (n = 6, X ± SD) *$p < 0.005$.

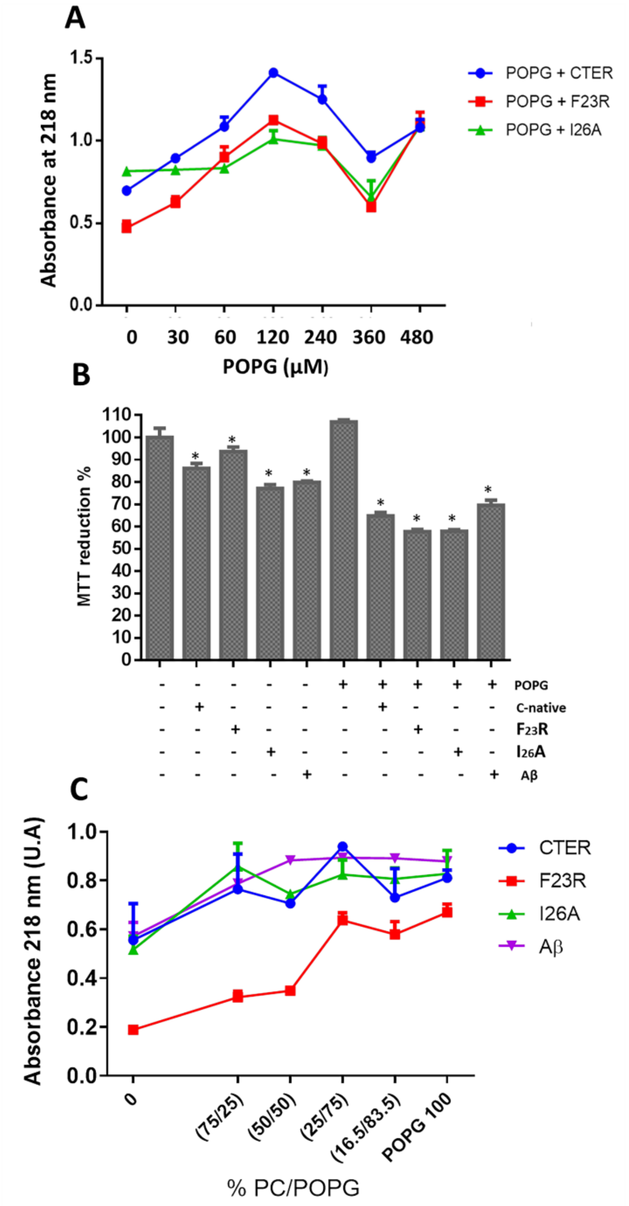

Supplementary Figure 8. Effect of lysophosphatidic acid (LPA) incubation on the secondary structure of C-native (**A**), F23R variant (**B**), and N-native (**C**), was evaluated by circular dichroism spectroscopy.

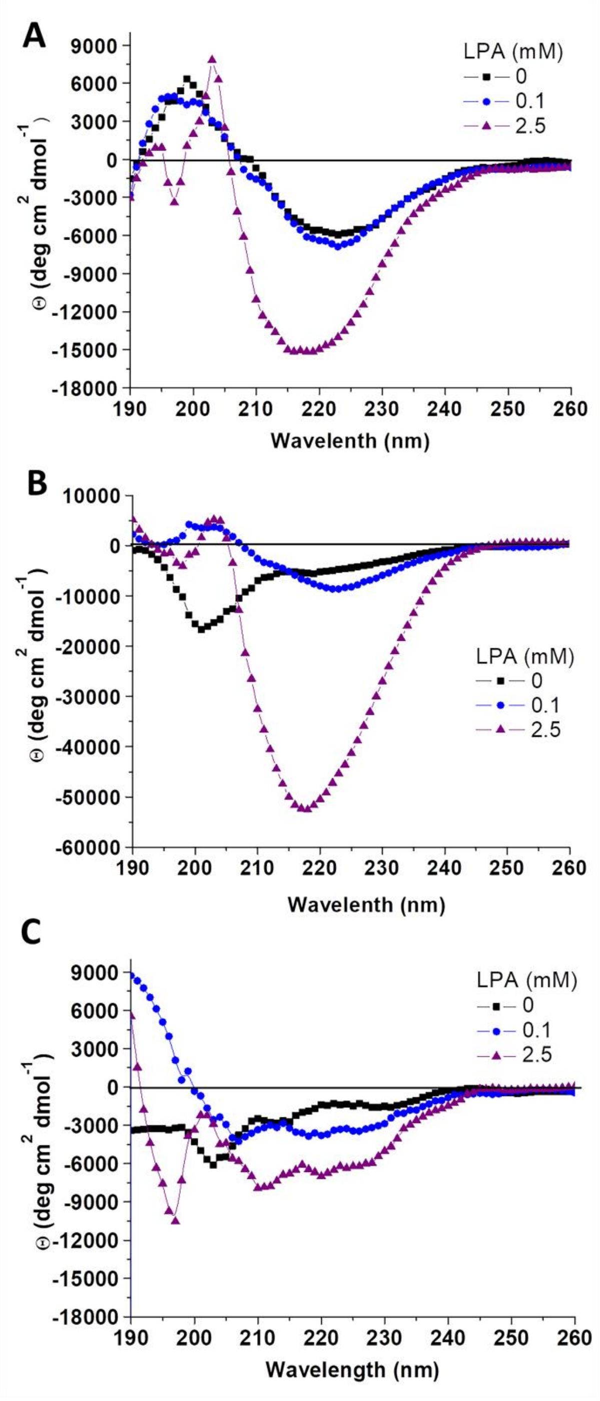

Supplementary Figure 9. Characterization of several fractions obtained during endoplasmic reticulum (ER) isolation. Three samples (1,2 and 3) corresponding to the total lysate, cytosol and ER were processed, and targets PDI, SERCA2, and β-actin were characterized. PVDF membranes were stained with Ponceau.

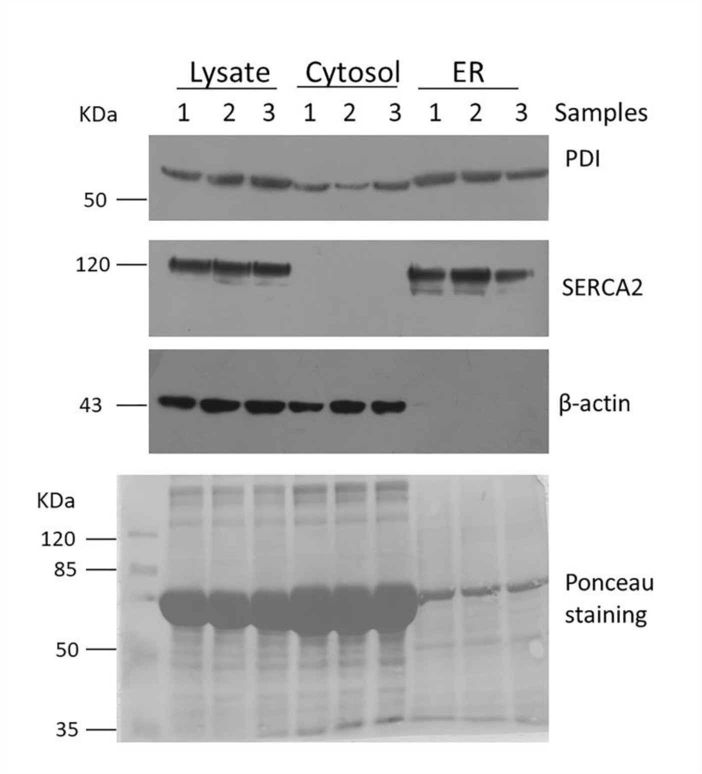

Supplement: Supplementary file 1 [file biomolecules-10-01201-s001.pdf]
